# Sex differences in activations to the sight of faces, scenes, body parts and tools in visual and non-visual cortical regions leading to the human hippocampus

**DOI:** 10.1186/s13293-026-00933-6

**Published:** 2026-06-01

**Authors:** Ruohan Zhang, Edmund T. Rolls, Jianfeng Feng

**Affiliations:** 1 Warwick Manufacturing Group, University of Warwick, Coventry, CV4 7AL UK; 2 Department of Computer Science, University of Warwick, Coventry, CV4 7AL UK; 3 Oxford Centre for Computational Neuroscience, Oxford, UK; 4 Institute of Science and Technology for Brain-Inspired Intelligence, Fudan University, 825 Zhangheng Road, Pudong New Area, Shanghai, 201203 China

**Keywords:** Human connectome, Sex differences, Human visual cortex, Activations to faces, places, body parts, and tools, Ventromedial visual cortical stream, Cortical scene regions

## Abstract

In a large Human Connectome Project dataset of 504 females and 452 males performing a 0-back memory task with visual images using the HCP-MultiModalParcellation, faces activated some key cortical regions more (using FDR correction) in females than males, including in the left hemisphere the fusiform face cortex and inferior temporal visual cortex TE2p; and visual motion regions in the MT+ complex. The sight of scenes activated more in males than females scene-related cortical regions including the medial parahippocampal scene (or place) regions such as PHA2 and earlier regions in the ventromedial cortical visual scene stream such as POS2, with differences found in both hemispheres. The sight of body parts activated more in females than males the inferior temporal visual cortex TE2p, some MT+ visual motion regions such as FST and PH, and also the perirhinal ‘What’ visual stream input to the hippocampal system via the perirhinal cortex, PeEc, in the left hemisphere. The sight of tools activated more in males than females and in the left hemisphere anterior temporal lobe regions such as TE2a, and regions that provide ‘What’ inputs to the hippocampal system, the lateral parahippocampal cortex and perirhinal cortex, PeEc. These cortical specialisations are likely to be relevant to behavioral differences for females compared to males in the processing of socially relevant stimuli such as faces and body parts; and to behavioral differences for males compared to females in the processing of spatial scenes and tools, with behavioral sex differences for scenes vs. faces present in these participants.

**Supplementary Information:**

The online version contains supplementary material available at 10.1186/s13293-026-00933-6.

## Plain language summary

Sex differences in neural processing are potentially important for translational neuroscience because epidemiological and meta-analytic evidence indicates robust sex-related differences in the prevalence, symptom profiles and clinical presentation of many neuropsychiatric and neurological disorders involving socio-emotional dysfunction. In this large-scale functional neuroimaging investigation with 956 participants with data obtained by the Human Connectome Project, we found that face-selective visual cortical regions in the inferior temporal visual cortex were more activated by viewing faces in females. Further, viewing body parts also activated nearby cortical regions more in females. In contrast, viewing spatial scenes activated the ventromedial visual cortical regions involved in scene perception more in males. Further, viewing tools also activated object-selective visual cortical regions more in males than females. These findings show that socially and emotionally relevant visual stimuli such as faces do produce different responses in the brains of females compared to males. These different sensitivities may be relevant to understanding sex differences in some key types of socio-emotional disorders such as depression.

**Supplementary Information:**

The online version contains supplementary material available at 10.1186/s13293-026-00933-6.

## Highlights

- In a large functional neuroimaging analysis with 956 participants, face-selective visual cortical regions in the inferior temporal visual cortex were more activated by viewing faces in females than males.
- Viewing body parts also activated nearby cortical regions more in females.
- Viewing spatial scenes activated the ventromedial visual cortical regions involved in scene perception more in males.
- Viewing tools also activated object-selective visual cortical regions more in males than females.
- These different sensitivities may be relevant to understanding sex differences in some key types of socio-emotional disorders such as depression.

**Supplementary Information:**

The online version contains supplementary material available at 10.1186/s13293-026-00933-6.

## Introduction

Biological sex is an important source of variability in human brain organization and cognition. Understanding sex-related differences is not only relevant for characterizing normative variation in brain structure and function, but also for clarifying why many neuropsychiatric conditions differ by sex in prevalence, age at onset, symptom profile, and clinical course [1, 2]. Prior work has shown that sex-related variation can be detected at multiple levels, including large-scale brain structure, structural connectivity, and functional responses during affective and cognitive tasks [3–9]. These findings indicate that sex should not be treated merely as a nuisance variable, but as a potentially informative dimension for understanding how human brain systems are organized and how they support behavior.

In the context of visual and socio-emotional processing, examining sex differences may be particularly informative because category-selective responses to socially relevant stimuli, such as faces and bodies, are shaped not only by perceptual demands but also by affective, mnemonic, and social-cognitive processes [5–11]. A more precise characterization of these differences may therefore help to link variation in visual cortical responses to broader biological and psychological mechanisms. Indeed, an important theoretical question is whether biological sex modulates category-selective visual processing in a domain-specific manner. Faces and bodies are socially salient categories that are closely linked to person perception and affective evaluation, whereas tools and places engage partially distinct perceptual and semantic systems. Testing whether sex-related effects differ across these categories may therefore help clarify whether such variation is broadly distributed across visual cortex or preferentially expressed in particular cortical regions for socially relevant stimulus classes.

The issue of sex differences in neural processing is also potentially important for translational neuroscience because substantial recent large-scale epidemiological and meta-analytic evidence indicates robust sex-related differences in the prevalence, symptom profiles and clinical presentation of many neuropsychiatric and neurological disorders involving socio-emotional dysfunction. For instance, major depressive disorder and anxiety-related conditions tend to be more prevalent in females across different populations, whereas conditions such as autism spectrum disorder are more commonly diagnosed in males [12, 13]. These differences extend beyond prevalence to include symptom expression, comorbidity patterns and treatment response. This suggests that biological and neurocognitive factors related to sex may play a role in shaping disorder-specific phenotypes [14, 15]. For example, increased amygdala responsiveness to negative stimuli including negative face expressions is postulated as a mechanism underlying the aberrant emotional processing in depressed individuals, manifesting as a negative bias in the perception and recognition of emotional stimuli [16–18].

From a systems neuroscience perspective, these observations suggest that sex-related variation in brain organisation, particularly within neural systems that support perception, emotion and social cognition, may contribute to differential vulnerability and behavioural expression among individuals. Examining whether sex modulates category-selective cortical responses to socially and perceptually relevant visual stimuli, such as faces, body parts, scenes and tools, could help to bridge the gap between population-level clinical findings and underlying neural mechanisms.

In this context, the aim of the research described here is to analyze whether there are sex differences in the activations to the sight of different types of visual stimuli, namely faces, scenes, body parts, and tools, in visual and also in non-visual cortical regions in humans. There is previous evidence that for example males may (on average, in a large group) have greater specialisation of some cortical regions for spatial stimuli such as scenes and for motor function, and that females may have greater specialisation of some cortical regions for verbal, reward-related and social stimuli [7, 19–23]. But with visual stimuli of the type used here, the activations may extend beyond visual cortical regions to for example somatosensory cortex, and even to visual motion areas when action-related visual stimuli such as tools are shown as stationary images, so that effects of the stimuli that reflect their meaning and use, that is, their semantic associations, can be investigated [24]. In this research, we were able to examine any possible female – male differences in the activation to stationary images of faces, places, body parts and tools in a large population of 956 individuals in the Human Connectome Project analysed in a 0-back working memory task which ensured that the visual stimuli were processed, but which had only a small memory requirement so that cortical responses to the visual stimuli could be analyzed. An additional aim of this research is to add function, by using task-related fMRI using visual stimuli, to the maps of cortical connectivity using the Human Connectome Project Multimodal Parcellation atlas HCP-MMP1 ^25^ that have been generated using effective and functional connectivity in the resting state, and diffusion tractography [26–30], with Human Connectome Project data [31, 32].

The HCP-MMP1 ^25^ is a well-founded parcellation of the human cerebral cortex into 180 cortical regions in each hemisphere because it utilises evidence from anatomy (cortical thickness and cortical myelin), functional connectivity, and task-related fMRI in 7 different tasks. This atlas provides a reference system that could be used in many investigations of human cortical function, to provide a reference standard to enable findings from different investigations to be compared 33. The HCP-MMP1 ^25^ has been extended to include 66 subcortical areas [34]. In addition, the connectivity and functions of most regions defined in the HCP-MMP atlas have been measured and assessed, and provide a foundation for understanding the systems-level organization and computations performed by the human brain [20, 24, 26–30, 33, 35–48]. For the above reasons, the HCP-MMP1 is the best cortical atlas we know for delineating the smallest cortical regions that can be reliably identified in humans, which may be building blocks of cortical function and provide a basis for advancing our understanding of cortical function [30]. It contrasts with many earlier parcellations of the cerebral cortex that are less computationally useful as they are based on gross topology [49, 50], or on cortical regions categorised primarily by functional connectivity [51].

Maps of cortical connectivity have been generated for many cortical systems using this HCP-MMP1 atlas using effective connectivity and functional connectivity in the resting state, and diffusion tractography. Effective connectivity measures the connectivity in each direction between each pair of brain regions by using time delays [27], and was complemented by measurement of functional connectivity, which given that it is based on Pearson correlations, can provide evidence about interactions between brain regions, but not about the direction or causality of effects [35, 36]. These methods were complemented by diffusion tractography which can measure direct connections between brain regions though not about the direction of connections [35], [37]. These three types of connectivity maps for the human cerebral cortex have been generated for the visual cortical regions [28, 40, 46]; the posterior parietal cortex [42]; the orbitofrontal cortex, anterior cingulate cortex, and ventromedial prefrontal cortex [35]; the posterior cingulate and medial parietal cortex [38]; the auditory cortex [39]; the amygdala compared to the orbitofrontal cortex [41]; the prefrontal and somatosensory cortex [29]; the frontal pole cortex [45]; and the hippocampal memory system [27, 36, 37].

These effective and functional connectivity maps of the 360 regions in the HCP-MMP1 atlas were generated with resting state fMRI, which because a task is not being performed may help to provide a foundation for understanding the underlying connectivity of the brain. But it is necessary to link the connectivity maps of the human brain to the functions of each cortical region, in order to understand better the flow of information through the brain, by providing evidence about the functions in which each cortical region is involved.

In the present research, we therefore measured separately in females and males the activations of each of the 360 cortical regions in the HCP-MMP1 atlas in task-related fMRI to different types of visual stimuli, which were stationary views of faces, scenes, body parts, and tools, in data collected for 956 participants by the HCP [31, 32] that were analysed here. All the data were in the surface-based version of the HCP-MMP1 atlas, as that provides the most accurate identification of each cortical region [25]. In order to identify cortical regions differently activated in females vs. males by each of faces, scenes, body parts, and tools, we measured the activations to each of these stimulus types compared to the mean activation averaged across all four types of visual stimuli. Although there has been much previous research on human brain activations to faces [52–55], scenes [24, 43, 44, 56–59], body parts [46, 54, 60–66], and tools [24, 67, 68] etc., we note that the aim of the research here is different, namely to measure the selective activations in females compared to males using the regions defined in the HCP-MMP1 atlas, partly because this atlas provides a well-founded framework for specifying cortical regions and comparing results between investigations, and importantly to add function to the connectivity maps for the human connectome referred to above. This aim is important for building a framework for better understanding human cerebral cortex function in health and in disease [30]. To enable comparison with previous investigations when the findings are presented here, investigations using the HCP-MMP1 atlas are cited where possible, so that the same cortical regions analyzed in different investigations can be directly compared.

## Methods

### HCP task and working memory paradigm

**Fig. 1:**
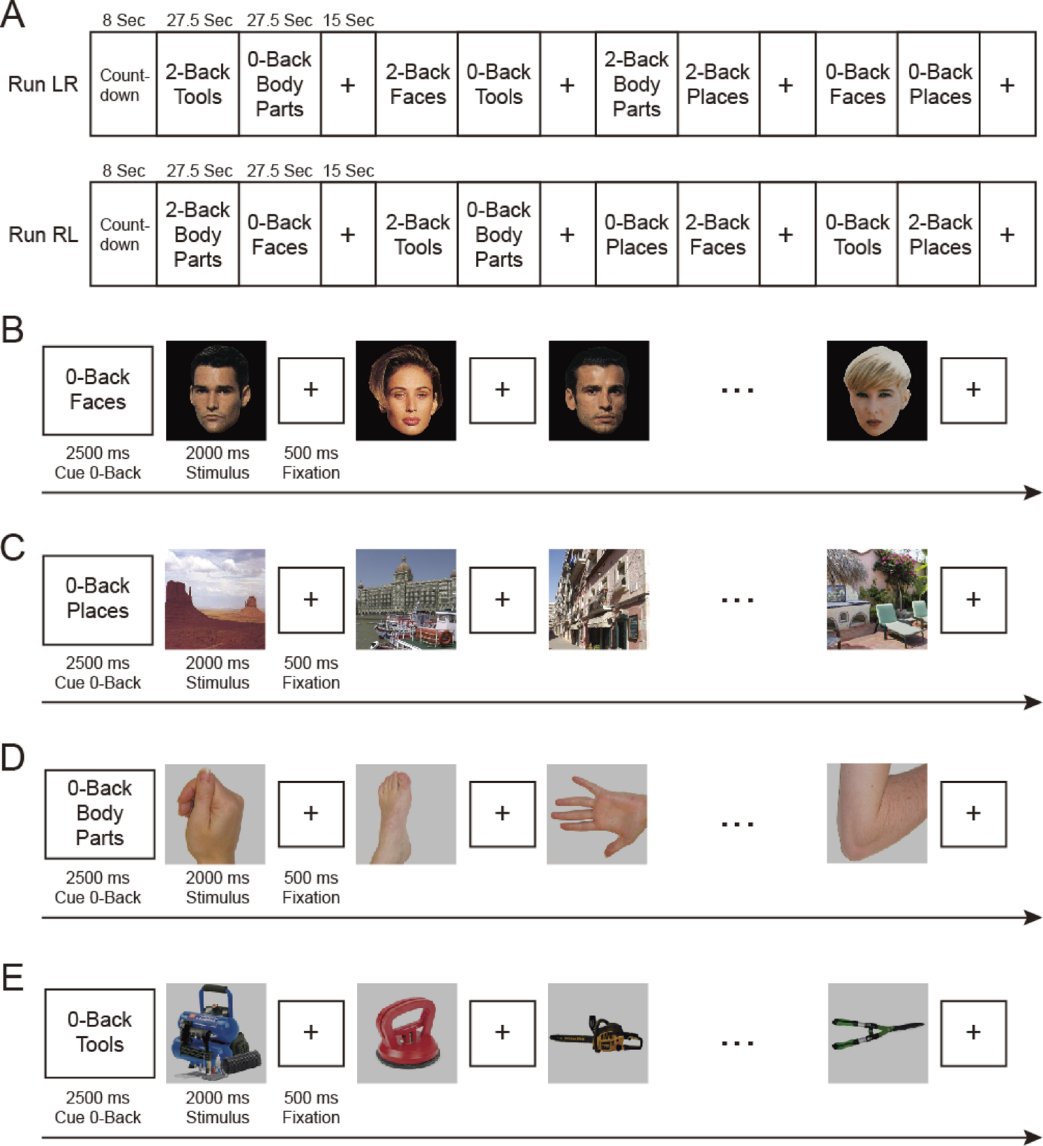
The Human Connectome Project Working Memory task for the 0-back condition [32]. Four stimulus types were used in a block design, faces, places, tools, and body parts. + indicates a fixation cross presented in the inter-trial interval. Examples of the large set of stimuli used are shown in this figure. In the 0-back condition used for most of the analyses described here, a target cue was presented at the start of each block in the cue period, and the participant had to respond ‘target’ to any presentation of that stimulus in the block. There were 2 runs in which data were acquired, and each run included 8 task blocks, 4 task blocks for 0-back, and 4 task blocks for 2-back. Each stimulus type (faces, scenes etc.) thus had 20 trials as 0-back, and 20 trials as 2-back. **A**. The task design in which runs of a task such as the 0-back task were performed. Each run consisted of a 2.5 s cue period followed by 10 trials in which a stimulus was shown for 2 s followed by a 0.5 s fixation period. The 10 stimuli in each run were thus presented over a 25 s period. Each run consisted of either faces, or places or body parts or tools. On 50% of runs, 0-back faces, places and tools were preceded by a 15 s screen showing only a fixation cross. **B**-**E**. Examples of the different 0-back runs

The Human Connectome Project (HCP) dataset provides task functional magnetic resonance imaging (fMRI) data for 7 cognitive tasks, one of which is the working memory task [32] which provided the data analysed here. In the working memory task, participants were presented with separate task blocks of trials for faces, places, body parts and tools [32]. The analyses described here were on the 0-back version of the task, illustrated in Fig. 1. The ‘face’ stimuli were as illustrated in Fig. 1. The ‘place’ stimuli were views of scenes as illustrated in Fig. 1, and are termed ‘scene stimuli’ here. (Details of the task, and the stimuli used, are available at https://www.humanconnectome.org/hcp-protocols-ya-task-fmri and https://balsa.wustl.edu/project?project=HCP_YA. The task involved visually presented category-specific stimuli as just described, and relevant details of stimulus timing, block structure, and acquisition protocol follow the standardized HCP task-fMRI design.) Within each task block, first an instruction image was presented for 2.5 s to indicate the stimulus task type and whether that block was 0-back or 2-back. Then 10 trials were run for a given stimulus type, with each stimulus shown for 2.0 s followed by an interstimulus interval of 0.5 s in which a cross was shown. The 10 stimuli in each block thus lasted for 25 s. In the analyses described here, the activations were measured as described below during these 25 s periods, which with a TR of 0.72 s provided 35 volumes. There were 2 runs in which data were acquired, and each run included 8 task blocks, 4 task blocks for 0-back, and 4 task blocks for 2-back. Each stimulus type (faces, scenes, body parts, and tools) thus had 20 trials as 0-back, and 20 trials as 2-back.

### HCP data acquisition

Functional magnetic resonance images (fMRI) were acquired from a large cohort of individuals participating in the working memory task of the HCP [32]. The data were obtained from the publicly available S1200 release (last updated: April 2018) of the HCP [69]. Participants provided written informed consent, and the scanning protocol was approved by the Institutional Review Board of Washington University in St. Louis, MO, USA (IRB #201204036). In this study, we utilized the task-based fMRI data of the working memory task from all 956 participants who completed both runs of the task with data quality approved by the HCP and who had covariates available.

The whole-brain EPI acquisitions were performed using a 32-channel head coil on a modified 3 T Siemens Skyra scanner. The imaging parameters included a TR of 720 ms, TE of 33.1 ms, flip angle of 52 degrees, bandwidth of 2290 Hz/Px, and in-plane FOV of 208 × 180 mm. Each functional volume comprised 72 slices with a voxel size of 2.0 mm isotropic. A multi-band acceleration factor of 8 was used during image acquisition [70, 71]. Two runs of each task were acquired, one with right-to-left phase encoding and the other with left-to-right phase encoding [32].

### Calculation of differences in activation for females – males

The current study employed surface-based timeseries data from the HCP for the working memory task. We parcellated the timeseries data into the 360 cortical regions defined by the surface-based HCP-MMP atlas (Glasser et al., 2016), with the brain regions and their labels shown in Fig. S1. We extracted the timeseries for each task block (e.g. 0-back faces as illustrated in Fig. 1B) which lasted for 27.5 s, using the timing information for each block provided by the HCP (https://www.humanconnectome.org/hcp-protocols-ya-task-fmri) (see Fig. 1). Within each task block, the BOLD signal showed a consistently high level to the set of stimuli in that task block for the last 20 timepoints in a block (with TR = 0.72 s) (see Fig. S3B of ^24^), and that period was used for the analysis of the responses to the visual stimuli. The calculation of the average BOLD signal level for each cortical region for each stimulus type for 0-back was averaged for each subject across the two runs.

For differences between females and males in the BOLD signal produced by the stimuli, as described above, the BOLD signal for each of the 180 cortical regions in each hemisphere was calculated for each participant from the last 20 timepoints (with TR = 0.72 s) in the 27.5 s period in which one stimulus type was being presented for the 0-back conditions (see Fig. 1). To test for sex-related differences in cortical responses for the 504 females and 452 males in the sample, we fitted region-wise general linear models with sex as the predictor of interest and age, education, drinking status, smoking status, handedness, and head motion as covariates of no interest. These covariates were included because they are known to influence BOLD signal estimates, individual differences in cognition, or data quality, and thus may contribute to between-subject variability unrelated to the effect of primary interest. In more detail, age and education were included as covariates to account for demographic and cognitive background differences, while drinking status and smoking status were included because lifestyle factors may contribute to between-subject variability in brain function. Head motion was included to reduce the influence of imaging-related artefact on BOLD estimates. We did not include all available HCP variables, but rather a restricted set of covariates chosen for conceptual relevance and model parsimony. This approach allowed us to estimate group differences while adjusting for potential confounding effects of demographic and imaging-related variables. The statistical inference for sex effects was thus based on the fitted general linear models.

In summary, the original HCP task-fMRI data were preprocessed and modeled within the standardized HCP pipeline. In the present study, we did not re-estimate voxel-level task GLMs from raw time series. Instead, we used the released HCP task-fMRI outputs and summarized task-related signals at the cortical parcel level using the HCP-MMP1 parcellation [25]. Accordingly, the inferential analyses reported here concern parcel-level estimates derived from HCP task-fMRI outputs rather than a de novo voxel-wise analysis.

**Fig. 2:**
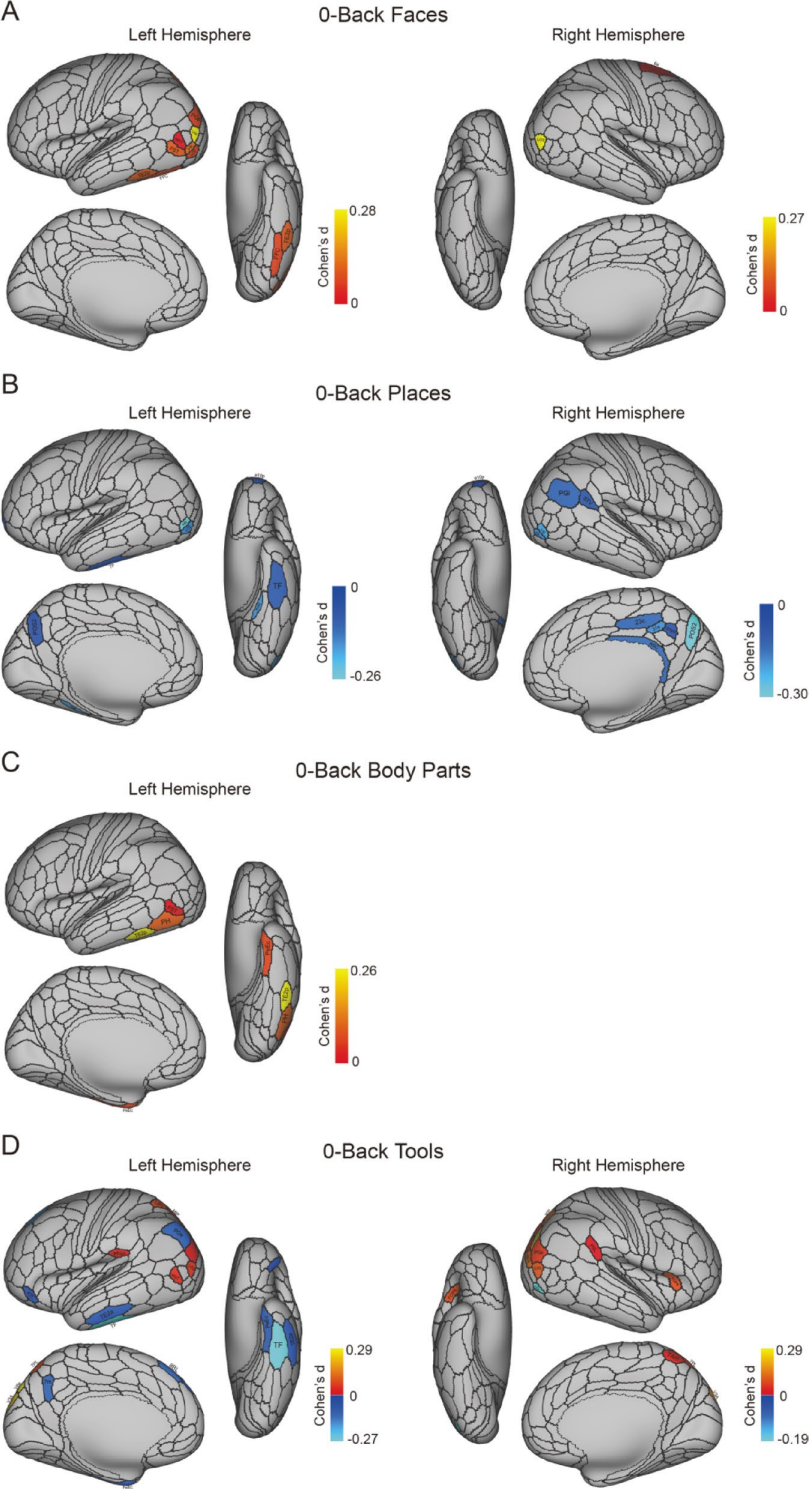
Brain regions in the left and right hemispheres with significant differences for females – males in the average BOLD signal for the faces, places (i.e. scenes as illustrated in Fig. 1), body parts, and tools) measured in the last 20 timepoints within each task block (see Methods). Positive values (red to yellow) thus represent greater signal in females, and negative values (blue) represent greater signal in males after FDR correction for multiple comparisons. (No significant differences were found in the right hemisphere for body parts.) The colorbar shows the magnitude of the differences expressed in terms of Cohen’s d, the effect size

To control for multiple comparisons across cortical regions, false discovery rate (FDR) correction [72] was applied, and the FDR-corrected results are reported in the main text. The effect size with Cohen’s d (the number of standard deviations between the means of the two groups [73]) was also measured, as this can be useful in interpreting the results beyond what is available only in probability values [74, 75]. For each significant region, we report the estimated effect size (Cohen’s d), together with the corresponding t-values, uncorrected p-values, and FDR-corrected p-values. The number of cortical regions included in the multiple-comparison procedure is defined by the HCP-MMP1 parcellation (180 regions per hemisphere), and FDR correction was applied across these tests. The findings with this analysis are described in the Results section in connection with Fig. 2.

### Calculation of the mean activation for each cortical region for females and males for each stimulus type

**Fig. 3:**
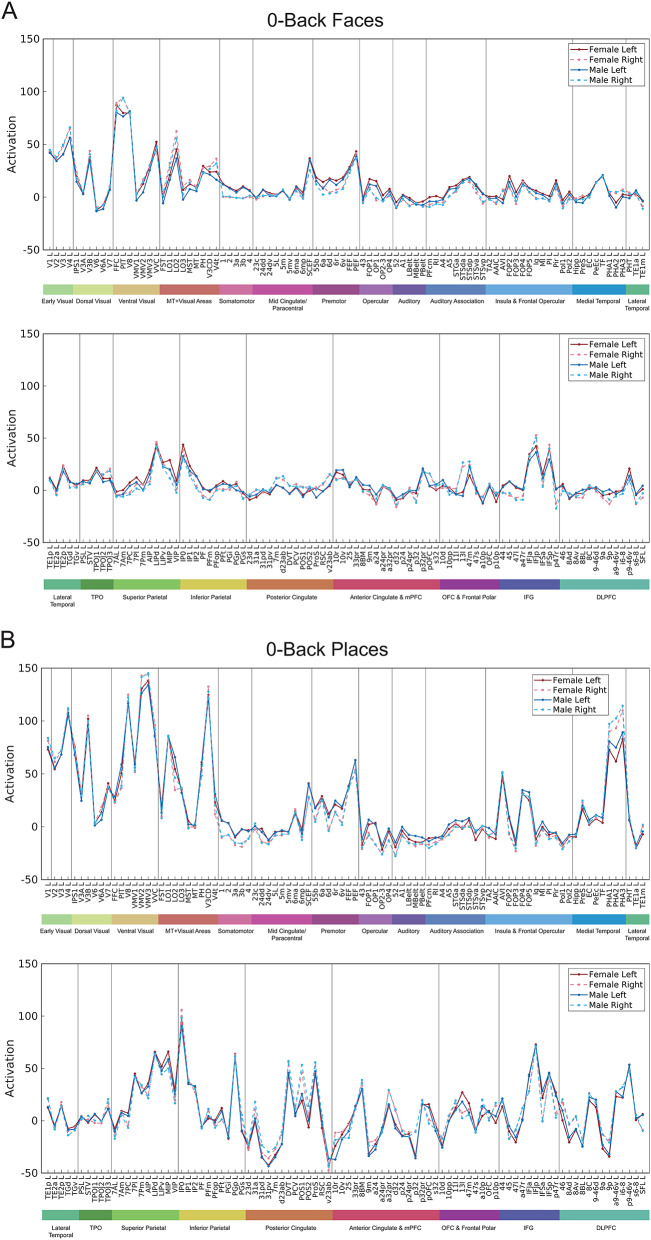

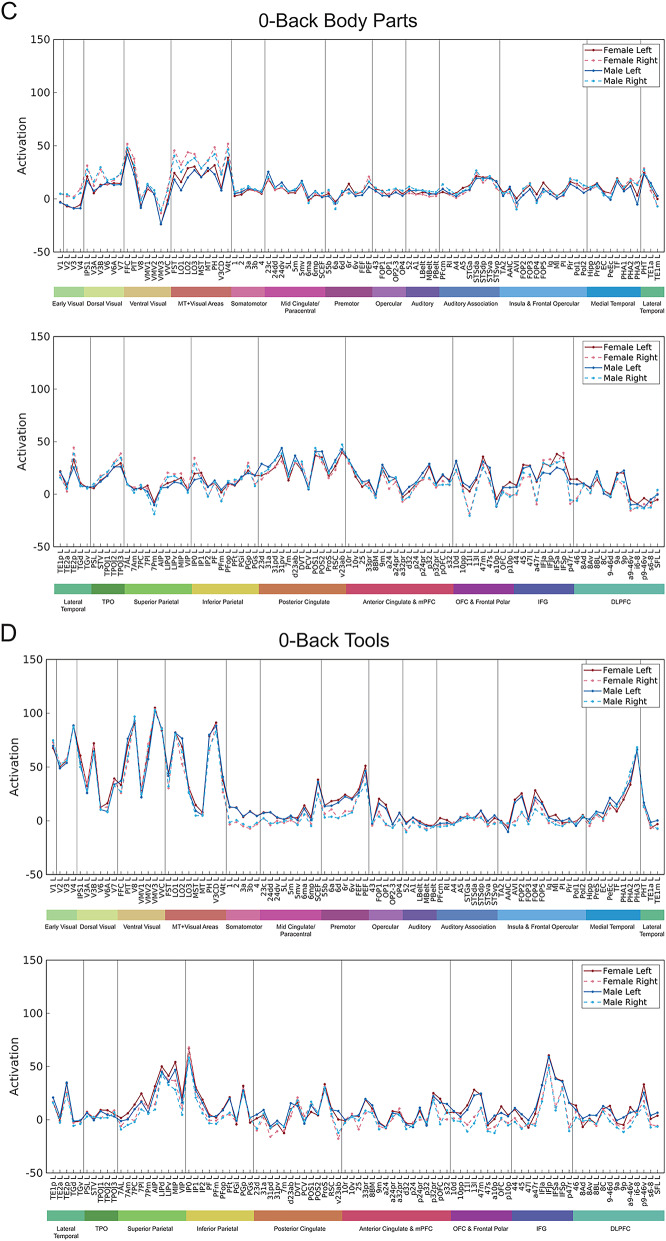
A. Activations to the sight of faces in females and males in the left and right hemispheres in 956 Human Connectome Project participants. The activations were measured as the % change from the BOLD signal between the first 15 timepoints before the BOLD signal had responded to the stimuli, to the BOLD signal in the last 20 timepoints within the 0-back blocks (when the BOLD signal response to the visual stimuli was occurring) for each of the four stimulus types (A faces; B places; C body parts; D tools). Negative values reflect some small deactivations in some cortical regions to each stimulus type (face, scenes, etc.). The upper plot is for the first 90 cortical regions for a hemisphere in the HCP-MMP atlas, and the lower plot is for the second 90 cortical regions, with the full names of each cortical region in the Supplementary Material. (Fig. 3A.eps). **B**. Activations to the sight of places (i.e. scenes as illustrated in Fig. 1) in females and males in the left and right hemispheres in 956 Human Connectome Project participants. Conventions as in Fig. 3A. (Fig3B.eps). **C**. Activations to the sight of body parts in females and males in the left and right hemispheres in 956 Human Connectome Project participants. Conventions as in Fig. 3A. (Fig3C.eps). **D** Activations to the sight of tools in females and males in the left and right hemispheres in 956 Human Connectome Project participants. Conventions as in Fig. 3A

To complement the statistical analysis just described, we also calculated the mean activation for each cortical region for females and males for each stimulus type, with the aim of displaying whether sets of related cortical regions showed similar differences between females and males. The measure of activation used here in a General Linear Model with the covariates regressed out is the BOLD signal in the last 20 timepoints with TR = 0.72 s during which each type of stimulus was shown (see Fig. 1) and there was an increase of BOLD signal in responsive regions, minus the BOLD signal in the first 15 timepoints before the activations had developed (see Fig. S3B of ^24^). The findings with this complementary analysis are described in the Results section in connection with Fig. 3.

### Correlations of the activations with cognitive measures

To help interpret the sex differences in activations to the visual stimuli, correlations with two a priori measures of relevant performance for these participants were analyzed.

In the Emotion Processing task, participants are presented with blocks of trials that either ask them to decide which of two faces presented on the bottom of the screen match the face at the top of the screen, or which of two shapes presented at the bottom of the screen match the shape at the top of the screen. The faces have either an angry or fearful expression. The measure of performance was the accuracy in the task.

The second measure of performance was the accuracy in the place condition for the working memory task.

## Results

### Female – male cortical activation differences by the sight of faces

Figure 2A shows the cortical regions with significant differences in activation for the contrast females – males to the sight of faces in each hemisphere. Red in the Figure indicates significantly higher activations of a cortical region in females, and blue indicates significantly higher activations in males, using FDR correction for multiple comparisons. The colorbar shows the magnitude of the differences expressed in terms of Cohen’s d, the effect size. The statistical values for the results shown in Fig. 2 are provided in Tables 1 and 2. In the left hemisphere, significantly higher activations to faces are found in females than males in the fusiform face cortex FFC and the closely related posterior inferior temporal visual cortex region TE2p (Fig. 2A). But interestingly, activations are also higher in females in some movement-related visual cortical regions in the MT+ visual cortical division, including MST, FST, V4t, LO2 and LO3, even though these were stationary visual images of faces. Activations were also significantly higher in females in the parietal cortex visuo-motor/spatial regions MIP and PGp. In the right hemisphere, significantly higher activations were found in only LO3 and 6a, indicating that it is the left hemisphere in females that shows the main differences in activations to the sight of faces.

**Table 1:** Cortical regions in the left hemisphere with significant differences for females – males in the average BOLD signal for the four visual stimuli for the last 20 timepoints

Regions	t Value	Cohen’s d	*p* Value	FDR
**0-Back Faces**				
LO3 L	4.36	0.28	1.47E-05	2.45E-03
IP0 L	4.22	0.27	2.72E-05	2.45E-03
V4t L	3.60	0.23	3.35E-04	0.0201
FST L	3.43	0.22	6.32E-04	0.0227
TE2p L	3.44	0.22	6.04E-04	0.0227
FFC L	3.26	0.21	1.16E-03	0.0243
LO2 L	3.28	0.21	1.09E-03	0.0243
MIP L	3.36	0.22	8.10E-04	0.0243
PGp L	3.25	0.21	1.21E-03	0.0243
MST L	3.03	0.20	2.49E-03	0.0448
**0-Back Places**				
V4t L	−4.03	−0.26	5.98E-05	0.0108
LO2 L	−3.57	−0.23	3.81E-04	0.0229
PHA2 L	−3.64	−0.24	2.89E-04	0.0229
TF L	−3.25	−0.21	1.18E-03	0.0426
a10p L	−3.29	−0.21	1.03E-03	0.0426
POS2 L	−3.16	−0.20	1.61E-03	0.0482
**0-Back Body Parts**				
TE2p L	3.94	0.26	8.70E-05	0.0157
PH L	3.67	0.24	2.61E-04	0.0203
PeEc L	3.60	0.23	3.39E-04	0.0203
FST L	3.52	0.23	4.58E-04	0.0206
**0-Back Tools**				
V6A L	4.40	0.29	1.20E-05	0.0022
TF L	−4.23	−0.27	2.54E-05	0.0023
V3A L	4.04	0.26	5.87E-05	0.0032
IP0 L	3.99	0.26	7.13E-05	0.0032
V3B L	3.66	0.24	2.62E-04	0.0094
8Ad L	−3.60	−0.23	3.35E-04	0.0101
IPS1 L	3.18	0.21	1.52E-03	0.0342
8BL L	−3.18	−0.21	1.52E-03	0.0342
7Pl L	3.04	0.20	2.45E-03	0.0365
LIPv L	3.05	0.20	2.37E-03	0.0365
MIP L	3.02	0.20	2.59E-03	0.0365
PGs L	−3.03	−0.20	2.51E-03	0.0365
7 m L	−3.02	−0.20	2.63E-03	0.0365
LO3 L	2.95	0.19	3.29E-03	0.0389
PeEc L	−2.93	−0.19	3.46E-03	0.0389
TE2a L	−2.96	−0.19	3.14E-03	0.0389
MST L	2.88	0.19	4.02E-03	0.0426
PFcm L	2.83	0.18	4.70E-03	0.0470
V7 L	2.77	0.18	5.75E-03	0.0487
PGp L	2.76	0.18	5.95E-03	0.0487
47 L L	−2.76	−0.18	5.84E-03	0.0487
9p L	−2.78	−0.18	5.58E-03	0.0487

**Table 2:** Cortical regions in the right hemisphere with significant differences for females – males in the average BOLD signal for the four visual stimuli for the last 20 timepoints

Regions	t Value	Cohen’s d	*p* Value	FDR
**0-Back Faces**				
LO3 R	4.23	0.27	2.56E-05	0.0046
6a R	3.49	0.23	5.07E-04	0.0456
**0-Back Places**				
V4t R	−4.00	−0.26	6.80E-05	0.0061
LO2 R	−3.78	−0.24	1.68E-04	0.0101
31a R	−3.69	−0.24	2.40E-04	0.0108
23c R	−3.37	−0.22	7.73E-04	0.0227
PGi R	−3.34	−0.22	8.83E-04	0.0227
RSC R	−3.38	−0.22	7.65E-04	0.0227
STV R	−3.09	−0.20	2.09E-03	0.0471
a10p R	−3.04	−0.20	2.46E-03	0.0492
31pd R	−3.00	−0.19	2.76E-03	0.0497
**0-Back Tools**				
IP0 R	4.44	0.29	9.87E-06	0.0018
V3A R	3.82	0.25	1.42E-04	0.0127
V3CD R	3.62	0.23	3.05E-04	0.0148
MIP R	3.60	0.23	3.29E-04	0.0148
LO3 R	3.35	0.22	8.39E-04	0.0302
FOP4 R	3.24	0.21	1.23E-03	0.0370
7Pl R	3.16	0.20	1.61E-03	0.0414
PGp R	3.09	0.20	2.04E-03	0.0459
IPS1 R	3.00	0.19	2.77E-03	0.0473
V3B R	3.01	0.19	2.71E-03	0.0473
LO2 R	−2.98	−0.19	2.93E-03	0.0473
7Am R	2.96	0.19	3.16E-03	0.0473
PSL R	2.90	0.19	3.79E-03	0.0487
VIP R	2.92	0.19	3.63E-03	0.0487

### Female – male cortical activation differences by the sight of scenes

It is shown in Fig. 2B (and Tables 1 and 2) that there are higher activations in males than females in some key regions implicated in representing spatial scenes, in particular the medial parahippocampal gyrus region PHA2, and region POS2 which is in an earlier part of the ventromedial cortical scene pathway [24, 28, 43, 44, 46] in the left hemisphere, and the posterior cingulate cortex regions RSC, 31a and 31pd, which have strong connectivity with the hippocampal system [38], in the right hemisphere. In the right hemisphere, parietal region PGi which is related to the spatial scene system [76] has higher activations in males. Further evidence for greater activation in males of spatial scene regions PHA2, PHA3, RSC, and POS2 in the right hemisphere is referred to in the Discussion. These greater activations to scenes in some scene areas in males in the right hemisphere are consistent with some lateralization of the spatial scene system to the right hemisphere [76]. In addition, regions V4t and LO2 in both hemispheres show higher activations to scenes in males. The lateral parahippocampal gyrus region TF also shows somewhat higher activation to scenes in males (in the left hemisphere).

### Female – male cortical activation differences by the sight of body parts

The sight of images of body parts produced significantly higher activations in females in the left hemisphere in posterior inferior temporal cortex region TE2p, and in MT+ visual motion related regions PH and FST (Fig. 2C; Table 1). In addition, there was higher activation in females to the sight of body parts in the perirhinal cortex PeEc, which connects ventrolateral visual cortical object regions with the hippocampal system [27, 28, 46, 77], in the left hemisphere.

### Female – male cortical activation differences by the sight of tools

The sight of tools produced significantly higher activation in males than females in an anterior temporal lobe visual object/semantic region TE2a [26, 28], and in regions connecting temporal lobe visual object/semantic regions to the hippocampal system, the lateral parahippocampal gyrus region TF, and the perirhinal cortex PeEc (Fig. 2D; Table 1) in the left hemisphere. Higher activations in males than females by tools was also found in inferior parietal PGs implicated in actions in space and connected with the anterior temporal lobe semantic system [26, 42], and in parietal 7 m involved in visuo-motor actions [42], in the left hemisphere (Fig. 2D).

In females vs. males, activations to tools were higher in some visual motion regions (V3A, V3B, V6A, IPSI, LO3, MST), inferior parietal PGp, and parietal LIPv, MIP, and IP0 (Fig. 2D; Tables 1 and 2). In females, activations to the sight of tools were also higher in some somatosensory cortical regions (FOP4, PFcm) [29] and in the PeriSylvian Language area PSL [26].

A possible implication is that in males, tools as objects and capable of performing actions in space are emphasized in the right hemisphere, and that in females tools as objects that move are emphasized in the left hemisphere.

### Cortical activation differences between females and males to visual stimuli illustrated for all cortical regions

The significant differences between activations in females and males to the sight of visual stimuli are shown in Fig. 2. However, to provide a more comprehensive overview for all cortical regions, the mean activations across the 956 HCP participants to the four types of visual stimuli, faces, scenes, body parts, and tools, are shown in Fig. 3, separately for the left and right hemispheres. The measure of activation used here is the BOLD signal in the last 20 timepoints with TR = 0.72 s during which each type of stimulus was shown (see Fig. 1) and there was an increase of BOLD signal in responsive regions, minus the BOLD signal in the first 15 timepoints before the activations had developed (see Fig. 3B of ^24^). In Fig. 3 it is possible to take advantage of what may be found in anatomically and functionally related cortical regions, rather than relying as in Fig. 2 on statistics performed individually on each of the large number (180) of cortical regions separately and then corrected for multiple comparisons.

Figure 3A shows how for faces, females tend to have higher activations than in males, in the left hemisphere especially, in visual face processing FFC; and visual motion-related PH and V4t, MIP, IP0; and somatosensory FOP1, OP1, OP2-3, PFcm, and FOP2; and inferior frontal language connected regions [26] IFJa and IFJp.

Figure 3B shows how for scenes (places), males tend to have higher activations than in females in the medial parahippocampal gyrus scenes-related PHA1-3 in both hemispheres, and also in POS1 and POS2 which are ventromedial visual cortical scene regions en route to PHA1-3 [24, 28]; and in the left hemisphere in some auditory cortical regions A1, LBelt, MBelt, PBelt; and superior temporal sulcus semantic regions STSva, STSda and STGa [26].

Figure 3C shows how for body parts, females tend to have higher activations than in males in inferior temporal visual cortex region TE2p; visual motion regions FST, LO1-3, PH, IP0 and IP1; in some somatosensory cortical regions FOP2-4; and inferior frontal gyrus language-related regions [26] IFJp, IFSa, IFSp.

Figure 3D shows how for tools, females tend to have higher activations than in males in some parietal visuo-motor regions 7Am, 7PC, 7PI, LIPv, and MIP; and that males have higher activations in some left hippocampus related regions TF, entorhinal cortex EC, and perirhinal cortex PeEc.

### Correlations of the activations with cognitive measures

To help interpret some of the sex differences in cortical activations to different visual stimuli described here, correlations of the activations of some of the key cortical regions with sex differences for faces and for scenes were analysed, as described in the Methods.

Two key cortical regions in which activations were higher in females than males during the 0-back face task were FFC and TE2p in the left hemisphere (Fig. 2A). It was found that these activations were correlated with the accuracy in the Emotion task (FFC *r* = 0.088, *p* < 0.007; TE2p *r* = 0.12, *p* = 0.0001). There was no significant correlation (*p* < 0.05) of the activations of these two regions with the performance in the scene condition of the working memory task.

A key cortical region in which activations were higher in males than females during the 0-back place (scene) task was the medial parahippocampal gyrus region PHA2 (Fig. 2B). It was found that these activations of PHA2 were correlated with the accuracy in the 0-back Place task (*r* = 0.17, *p* < 2e-7). There was no significant correlation (*p* < 0.05) of the activations of PHA2 with the performance in the Emotion task.

The reliability of this dissociation was confirmed in a ChiSquare analysis for the number of significant correlations found (*p* < 0.05) in which one factor was task (any Emotion task measure vs. any 0-back working memory task measure), and the other factor was cortical region (FFC + TE2p vs. PHA2), for which Chi-square = 17, *p* = 3.7e-05, df = 1.

These analyses show that the greater activations of FFC and TE2p in females for faces are relevant to behavior, for the activations of these cortical regions are correlated with performance on the Emotion task. Further, these analyses show that the greater activation of the medial parahippocampal cortical region PHA2 in males for places (scenes) is relevant to behaviour, for the activations of this cortical region are correlated with performance on the 0-back Place task.

Consistent with these results, the accuracy of the behavioral performance in the Emotion task (Emotion_task_Acc) was higher in the females (t = 1.97 df = 954, *p* < 0.05); and the accuracy in the behavioral performance of the 2-back working memory place task (WM_Task_2bk_Place_Acc) was higher in males than females (t=−3.72 df = 954, *p* = 0.0002). It was also consistent with the results presented here, that the females scored higher on a measure of fear affect (FearAffectUnadj) than males (t = 4.39 df = 954, *p* < 0.00002) given that in the Emotion task the faces had either an angry or fearful expression.

## Discussion

In this analysis of activations in a large dataset of 956 females and males produced by visual stimuli, it was found that faces activated some key brain regions in the left hemisphere more in females than males, including the fusiform face cortex and inferior temporal visual cortex TE2p; and visual motion regions in the MT+ complex. Faces are highly socially and emotionally relevant, and that may relate to these findings. The sight of spatial scenes activated more in males than females scene-related cortical regions including the medial parahippocampal scene (or place) regions such as PHA2 and earlier regions in the ventromedial cortical visual scene stream such as POS2, with differences in scene-related cortical regions found in both hemispheres. The sight of body parts activated more in females than males the inferior temporal visual cortex TE2p, some MT+ visual motion regions such as FST and PH, and also the perirhinal ‘What’ visual stream input to the hippocampal system via the perirhinal cortex, PeEc, with differences found in both hemispheres. The sight of tools activated more in males than females anterior temporal lobe regions such as TE2a, and regions that provide ‘What’ inputs to the hippocampal system, the lateral parahippocampal cortex region TF and perirhinal cortex, PeEc. These cortical specialisations are likely to be relevant to differences for females compared to males in the processing of socially relevant stimuli such as faces and body parts; and to differences for males compared to females in the processing of spatial scenes and tools, with behavioral differences found in the participants in this investigation. The relevance to behavior of these differences in the activations was also shown by correlations of key cortical activation differences for faces vs. scenes with the behavioral performance of females vs. males.

Greater activations were found in females than males to faces in cortical regions that included the fusiform face cortex and inferior temporal visual cortex TE2p; and visual motion regions in the MT+ complex, in the left hemisphere (Fig. 2A). The finding of greater activations in the FFC and TE2p in females to the sight of faces in the left hemisphere may relate to some specialisation for face identification and also face expression identification which is so important in social and emotional behavior in females [7, 9]. Indeed, in this cohort, the accuracy of the behavioral performance on the Emotion task in which faces were the visual stimuli was higher in females than males, as reported in the Results. Further examples are that females can interpret infant face expressions better than can males, and that females found baby faces more salient than did males [7, 9]. Although face processing is in some investigations greater in the right hemisphere [52], in the present investigation the activations in females were greater than in males in the left hemisphere, which is consistent with some other findings [7, 9]. The greater activation in females in the MT+ regions may relate to the greater salience of faces in females, and to their greater sensitivity to biological motion [7, 9].

The greater activations in females than males to body parts in perceptual regions such as the inferior temporal visual cortex TE2p (in both hemispheres) may also relate to differences in how females perceive body parts, with females perhaps more sensitive to the fact that some of the images used might indicate damaged limbs (see Fig. 1), or to the importance of perceiving bodies in females, and especially to the importance of body gestures in social behavior [7, 9]. Indeed, the latter may relate to the greater activations found in females in some of the MT+ visual motion-related cortical regions (Fig. 2C).

The greater activations in males than females to the sight of spatial scenes of scene-related cortical regions including the medial parahippocampal scene (or place) regions such as PHA2 and earlier regions in the ventromedial cortical visual scene stream such as POS2, with differences found in both hemispheres, may provide a basis for greater or different spatial and scene processing in males [21] (and of course this is just on average, with considerable overlap between females and males). Consistent with this, there is some evidence that spatial strategies may differ, with males attending more to geometric cues while females attend to landmark and visual cues [78]. Further, in this cohort, the accuracy of the behavioral performance on the 2-back place task in which spatial scenes were the visual stimuli was higher in males than females, as reported in the Results.

The greater activations in males than females to the sight of tools in anterior temporal lobe regions such as TE2a, and regions that provide ‘What’ inputs to the hippocampal system, the lateral parahippocampal cortex region TF and perirhinal cortex, PeEc, suggests specialisation of function not only in semantic regions in the anterior temporal lobe, but also in providing ‘What’ inputs about tools to the hippocampal episodic memory system.

It is emphasised that these cortical activation differences between females and males are likely to be relevant to differences in behavior to these visual stimuli between females and males. For example, not only was the performance on the Emotion task correlated with the greater activations in FFC and TE2p in females, and the performance on the place task correlated with the greater activations of PHA2 in the Place task, but also for these participants the accuracy of the behavioral performance on the Emotion task was higher in the females than males; and the accuracy in the behavioral performance of the Place task was higher in males than females, as reported in the Results.

In terms of the biological interpretation and significance of the female – male differences in processing visual stimuli that are described here, a possible interpretation is that sex-related differences in responses to face stimuli may reflect variation in neural systems supporting socially relevant visual processing, rather than a generic difference in “emotionality.” Face stimuli engage cortical networks that include ventral occipitotemporal cortex to the inferior temporal visual cortex involved in face identity processing, superior temporal cortex regions involved in the biological motion of face and body stimuli, and affective evaluation involving the orbitofrontal cortex and amygdala [28, 33, 46, 48, 79–81]. Prior studies have reported sex-related differences in aspects of face and emotion processing, including hemispheric asymmetry, stress-dependent modulation of fusiform responses, and neural responses during facial affect evaluation [6–11, 82]. Against this background, the present findings may be interpreted as evidence for modest sex-related variation in the neural processing of socially salient visual stimuli, rather than as proof of broad psychological traits. In addition, it is noted that many earlier studies relied on relatively small samples, task-specific contrasts, or region-of-interest analyses. The present study extends previous research by examining category-related cortical responses within a standardized whole-cortex parcellation framework in a large open-access dataset. Moreover, because our task design, contrasts, stimulus categories, and population size differ from those used in any earlier studies, the current results should be viewed as new and large-scale results for female – male difference in processing four types of visual stimuli, faces, scenes, tools, and body parts, and extending the analyses beyond the ventral cortical visual stream for object and face processing to further brain regions where the activations reflect the semantics of the stimuli[24, 76].

In relation to the processing of spatial scenes, the higher activations in males of spatial scene cortical regions may be related to processing differences between males and females, for males perform better at allocentric spatial processing than females, with little difference in egocentric spatial processing [83]. Thus the differences in neural processing of scenes in males described here may be related to especially certain forms of spatial processing, and not just to some general difference such as attention. (We further note that attention was required on every trial of the 0-back task, as a response had to be made on every trial.)

In the results reported here, total brain or gray matter volume were not regressed out as a covariate of no interest, for although this may be appropriate in structural studies of volume differences between females and males, there is no clear rationale for this when brain activations are being investigated as here; and when the activations in visual cortical face areas are higher in females and conversely in visual cortical scene areas are higher in males. However, when brain volume was regressed out as a covariate of no interest, key results, greater activations of scene areas by viewing scenes in males than in females, remained significant (right hemisphere, regions PHA2, PHA3, RSC, and POS2).

Although the brain differences in the activations described here were significant after FDR correction for multiple comparisons, the Cohen’s d values were not large (typically between 0.2 and 0.3, see Tables 1 and 2), but were comparable to a previous investigation of sex differences in the brain [20]. Indeed, a strength of the current investigation is that it did use a large sample size, of 956 participants, which is important as large sample sizes are needed for reliable neuroimaging investigations [84]. Further, an important conclusion is that although sex differences in brain functioning may be small, there are some significant differences, and they do relate to significant differences in behavior, as shown in this investigation.

Overall, the results described here provide clear and quantitative evidence that there are differences in how the brains of females vs. males respond to visual stimuli that include faces, scenes, body parts, and tools. The differences between females and males found are clearly very significant, and generally the effect sizes as shown by the Cohen’s d values shown in Fig. 2; Tables 1 and 2 were reasonable for neuroimaging investigations. This emphasises the utility of a large carefully curated dataset such as that provided by the Human Connectome Project [31, 32], which with the 956 participants included in the present investigation allow clear results to be obtained that relate to well-identified cortical regions from the HCP-MMP atlas [25] with different functions [30]. We do note that the observed effect sizes were small in magnitude according to conventional benchmarks for Cohen’s d [73–75], and should therefore be interpreted as subtle but statistically reliable group-level differences rather than large sex-specific dissociations. In this context, a key contribution of this investigation is the use of a large number of participants (956), which has enabled statistically reliable and not over-inflated effects to be discovered for differences between females and males. Indeed, these cortical activation differences between females and males are likely to be relevant to differences in behavior to these visual stimuli between females and males that were demonstrated in these participants. The results described here therefore make an important contribution to the understanding of the differences in the functioning of different parts of the female and male brain, extending importantly by the use of a large population size, the use of the HCP-MMP atlas, and the use of high quality HCP functional neuroimaging data, some previous investigations [7, 9, 19–21, 85].

## Supplementary Information

Supplementary Material 1

## Data Availability

Data and code availability. The data are available at the HCP website http://www.humanconnectome.org/. Standard Matlab functions were used to calculate the functional connectivity, to perform the regression analyses, and to perform the FDR corrections for multiple comparisons.

## References

[1] Andreano JM, Cahill L. Sex influences on the neurobiology of learning and memory. Learn Mem. 2009;16:248–66. 10.1101/lm.918309. 19318467 10.1101/lm.918309

[2] Cahill L. Why sex matters for neuroscience. Nat Rev Neurosci. 2006;7:477–84. 10.1038/nrn1909. 16688123 10.1038/nrn1909

[3] Ingalhalikar M, et al. Sex differences in the structural connectome of the human brain. Proc Natl Acad Sci U S A. 2014;111:823–8. 10.1073/pnas.1316909110. 24297904 10.1073/pnas.1316909110 PMC3896179

[4] Ruigrok AN, et al. A meta-analysis of sex differences in human brain structure. Neurosci Biobehav Rev. 2014;39:34–50. 10.1016/j.neubiorev.2013.12.004. 24374381 10.1016/j.neubiorev.2013.12.004 PMC3969295

[5] Briceno EM, et al. Age and gender modulate the neural circuitry supporting facial emotion processing in adults with major depressive disorder. Am J Geriatr Psychiatry. 2015;23:304–13. 10.1016/j.jagp.2014.05.007. 25085721 10.1016/j.jagp.2014.05.007 PMC4241383

[6] Weisenbach SL, et al. Reduced emotion processing efficiency in healthy males relative to females. Soc Cogn Affect Neurosci. 2014;9:316–25. 10.1093/scan/nss137. 23196633 10.1093/scan/nss137 PMC3980801

[7] Proverbio AM. Sex differences in the social brain and in social cognition. J Neurosci Res. 2023;101:730–8. 10.1002/jnr.24787. 33608982 10.1002/jnr.24787

[8] Proverbio AM, Brignone V, Matarazzo S, Del Zotto M, Zani A. Gender differences in hemispheric asymmetry for face processing. BMC Neurosci. 2006;7:44. 10.1186/1471-2202-7-44. 16762056 10.1186/1471-2202-7-44 PMC1523199

[9] Proverbio AM. Sex differences in social cognition. In: Boggio PS, editor. Social and Affective Neuroscience of Everyday Human Interaction: From Theory to Methodology. 2023. p. 85–106. 37988484

[10] Mather M, Lighthall NR, Nga L, Gorlick MA. Sex differences in how stress affects brain activity during face viewing. Neuroreport. 2010;21:933–7. 10.1097/WNR.0b013e32833ddd92. 20808182 10.1097/WNR.0b013e32833ddd92 PMC2948784

[11] Kret ME, De Gelder B. A review on sex differences in processing emotional signals. Neuropsychologia. 2012;50:1211–21. 10.1016/j.neuropsychologia.2011.12.022. 22245006 10.1016/j.neuropsychologia.2011.12.022

[12] Dai F, Cai Y, Chen M, Dai Y. Global trends of depressive disorders among women of reproductive age from 1990 to 2021: a systematic analysis of burden, sociodemographic disparities, and health workforce correlations. BMC Psychiatry. 2025;25:263. 10.1186/s12888-025-06697-4. 40114132 10.1186/s12888-025-06697-4 PMC11924784

[13] Kayrouz R, et al. A review of the 257 meta-analyses of the differences between females and males in prevalence and risk, protective factors, and treatment outcomes for mental disorder. BMC Psychiatry. 2025;25:677. 10.1186/s12888-025-06848-7. 40610965 10.1186/s12888-025-06848-7 PMC12224573

[14] Weiss SJ, et al. Gender differences in symptom profiles of individuals being treated for mood disorders. Journal of Mood & Anxiety Disorders. 2025;12:100152. 10.1016/j.xjmad.2025.100152. 41127122 10.1016/j.xjmad.2025.100152 PMC12538442

[15] Nolting IKL, Morina N, Hoppen TH, Tam KP, Kip A. A meta-analysis on gender differences in prevalence estimates of mental disorders following exposure to natural hazards. Eur J Psychotraumatol. 2025;16:2476809. 10.1080/20008066.2025.2476809. 40135376 10.1080/20008066.2025.2476809 PMC11948360

[16] Warren MB, Pringle A, Harmer CJ. A neurocognitive model for understanding treatment action in depression. Philos Trans R Soc Lond B Biol Sci. 2015;370:20140213. 10.1098/rstb.2014.0213. 26240428 10.1098/rstb.2014.0213 PMC4528825

[17] Godlewska BR, Browning M, Norbury R, Cowen PJ, Harmer CJ. Early changes in emotional processing as a marker of clinical response to SSRI treatment in depression. Transl Psychiatry. 2016;6:e957. 10.1038/tp.2016.130. 27874847 10.1038/tp.2016.130 PMC5314109

[18] Pringle A, Harmer CJ. The effects of drugs on human models of emotional processing: an account of antidepressant drug treatment. Dialogues Clin Neurosci. 2015;17:477–87. 10.31887/DCNS.2015.17.4/apringle. 26869848 10.31887/DCNS.2015.17.4/apringle PMC4734885

[19] Vosberg DE. Sex and gender in population neuroscience. Curr Top Behav Neurosci. 2024;68:87–105. 10.1007/7854_2024_468. 38509404 10.1007/7854_2024_468

[20] Zhang R, Rolls ET, Cheng W, Feng J. Different cortical connectivities in human females and males relate to differences in strength and body composition, reward and emotional systems, and memory. Brain Struct Funct. 2024;229:47–61. 10.1007/s00429-023-02720-0. 37861743 10.1007/s00429-023-02720-0 PMC10827883

[21] Gur RC, Gur RE. Complementarity of sex differences in brain and behavior: from laterality to multimodal neuroimaging. J Neurosci Res. 2017;95:189–99. 10.1002/jnr.23830. 27870413 10.1002/jnr.23830 PMC5129843

[22] Ritchie SJ, et al. Sex differences in the adult human brain: evidence from 5216 UK Biobank participants. Cereb Cortex. 2018;28:2959–75. 10.1093/cercor/bhy109. 29771288 10.1093/cercor/bhy109 PMC6041980

[23] Liu S, Seidlitz J, Blumenthal JD, Clasen LS, Raznahan A. Integrative structural, functional, and transcriptomic analyses of sex-biased brain organization in humans. Proc Natl Acad Sci U S A. 2020;117:18788–98. 10.1073/pnas.1919091117. 32690678 10.1073/pnas.1919091117 PMC7414084

[24] Rolls ET, Feng J, Zhang R. Selective activations and functional connectivities to the sight of faces, scenes, body parts and tools in visual and non-visual cortical regions leading to the human hippocampus. Brain Struct Funct. 2024;229:1471–93. 10.1007/s00429-024-02811-6. 38839620 10.1007/s00429-024-02811-6 PMC11176242

[25] Glasser MF, et al. A multi-modal parcellation of human cerebral cortex. Nature. 2016;536:171–8. 10.1038/nature18933. 27437579 10.1038/nature18933 PMC4990127

[26] Rolls ET, Deco G, Huang C-C, Feng J. The human language effective connectome. Neuroimage. 2022;258:119352. 10.1016/j.neuroimage.2022.119352. 35659999 10.1016/j.neuroimage.2022.119352

[27] Rolls ET, Deco G, Huang CC, Feng J. The effective connectivity of the human hippocampal memory system. Cereb Cortex. 2022;32:3706–25. 10.1093/cercor/bhab442. 35034120 10.1093/cercor/bhab442

[28] Rolls ET, Deco G, Huang C-C, Feng J. Multiple cortical visual streams in humans. Cereb Cortex. 2023;33:3319–49. 10.1093/cercor/bhac276. 35834308 10.1093/cercor/bhac276

[29] Rolls ET, Deco G, Huang CC, Feng J. Prefrontal and somatosensory-motor cortex effective connectivity in humans. Cereb Cortex. 2023;33:4939–63. 10.1093/cercor/bhac391. 36227217 10.1093/cercor/bhac391

[30] Rolls ET. Brain Computations and Connectivity. Open Access: Oxford University Press; 2023.

[31] Glasser MF, et al. The Human Connectome Project’s neuroimaging approach. Nat Neurosci. 2016;19:1175–87. 10.1038/nn.4361. 27571196 10.1038/nn.4361 PMC6172654

[32] Barch DM, et al. Function in the human connectome: task-fMRI and individual differences in behavior. Neuroimage. 2013;80:169–89. 10.1016/j.neuroimage.2013.05.033. 23684877 10.1016/j.neuroimage.2013.05.033 PMC4011498

[33] Rolls ET. Brain Computations and Principles; and AI. Open Access: Oxford University Press; 2026.

[34] Huang CC, Rolls ET, Feng J, Lin CP. An extended Human Connectome Project multimodal parcellation atlas of the human cortex and subcortical areas. Brain Struct Funct. 2022;227:763–78. 10.1007/s00429-021-02421-6. 34791508 10.1007/s00429-021-02421-6

[35] Rolls ET, Deco G, Huang CC, Feng J. The human orbitofrontal cortex, vmPFC, and anterior cingulate cortex effective connectome: emotion, memory, and action. Cereb Cortex. 2022;33:330–56. 10.1093/cercor/bhac070. 35233615 10.1093/cercor/bhac070

[36] Ma Q, Rolls ET, Huang C-C, Cheng W, Feng J. Extensive cortical functional connectivity of the human hippocampal memory system. Cortex. 2022;147:83–101. 10.1016/j.cortex.2021.11.014. 35026557 10.1016/j.cortex.2021.11.014

[37] Huang C-C, Rolls ET, Hsu C-C, Feng J, Lin C-P. Extensive cortical connectivity of the human hippocampal memory system: beyond the what and where dual-stream model. Cereb Cortex. 2021;31:4652–69. 10.1093/cercor/bhab113. 34013342 10.1093/cercor/bhab113 PMC8866812

[38] Rolls ET, Wirth S, Deco G, Huang C-C, Feng J. The human posterior cingulate, retrosplenial and medial parietal cortex effective connectome, and implications for memory and navigation. Hum Brain Mapp. 2023;44:629–55. 10.1002/HBM.26089. 36178249 10.1002/hbm.26089 PMC9842927

[39] Rolls ET, Rauschecker JP, Deco G, Huang CC, Feng J. Auditory cortical connectivity in humans. Cereb Cortex. 2023;33:6207–27. 10.1093/cercor/bhac496. 36573464 10.1093/cercor/bhac496 PMC10422925

[40] Rolls ET, Deco G, Zhang Y, Feng J. Hierarchical organization of the human ventral visual streams revealed with magnetoencephalography. Cereb Cortex. 2023;33:10686–701. 10.1093/cercor/bhad318. 37689834 10.1093/cercor/bhad318

[41] Rolls ET, Deco G, Huang C-C, Feng J. Human amygdala compared to orbitofrontal cortex connectivity, and emotion. Prog Neurobiol. 2023;220:102385. 10.1016/j.pneurobio.2022.102385. 36442728 10.1016/j.pneurobio.2022.102385

[42] Rolls ET, Deco G, Huang CC, Feng J. The human posterior parietal cortex: effective connectome, and its relation to function. Cereb Cortex. 2023;33:3142–70. 10.1093/cercor/bhac266. 35834902 10.1093/cercor/bhac266 PMC10401905

[43] Rolls ET, Zhang R, Deco G, Vatansever D, Feng J. Selective brain activations and connectivities related to the storage and recall of human object-location, reward-location, and word-pair episodic memories. Hum Brain Mapp. 2024;45:e70056. 10.1002/hbm.70056. 39436048 10.1002/hbm.70056 PMC11494686

[44] Rolls ET, et al. A ventromedial visual cortical ‘Where’ stream to the human hippocampus for spatial scenes revealed with magnetoencephalography. Commun Biol. 2024;7:1047. 10.1038/s42003-024-06719-z. 39183244 10.1038/s42003-024-06719-z PMC11345434

[45] Rolls ET, Deco G, Huang CC, Feng J. The connectivity of the human frontal pole cortex, and a theory of its involvement in exploit versus explore. Cereb Cortex. 2024;34:1–19. 10.1093/cercor/bhad416. 37991264 10.1093/cercor/bhad416

[46] Rolls ET. Two what, two where, visual cortical streams in humans. Neurosci Biobehav Rev. 2024;160:105650. 10.1016/j.neubiorev.2024.105650. 38574782 10.1016/j.neubiorev.2024.105650

[47] Rolls ET. Hippocampal, revolutions. Neurosci Biobehav Rev. 2026;180:106492. 10.1016/j.neubiorev.2025.106492. 41308965 10.1016/j.neubiorev.2025.106492

[48] Rolls ET. Neuroscience Discoveries. Open Access: MIT Press; 2026. 10.7551/mitpress/18504.001.0001.

[49] Rolls ET, Huang CC, Lin CP, Feng J, Joliot M. Automated Anatomical Labelling Atlas 3. Neuroimage. 2020;206:116189. 10.1016/j.neuroimage.2019.116189. 31521825 10.1016/j.neuroimage.2019.116189

[50] Rolls ET, Joliot M, Tzourio-Mazoyer N. Implementation of a new parcellation of the orbitofrontal cortex in the automated anatomical labeling atlas. NeuroImage. 2015;122:1–5. 10.1016/j.neuroimage.2015.07.075. 26241684 10.1016/j.neuroimage.2015.07.075

[51] Power JD, et al. Functional network organization of the human brain. Neuron. 2011;72:665–78. 10.1016/j.neuron.2011.09.006. 22099467 10.1016/j.neuron.2011.09.006 PMC3222858

[52] Kanwisher N, McDermott J, Chun MM. The fusiform face area: a module in human extrastriate cortex specialized for face perception. J Neurosci. 1997;17:4302–11. 10.1523/JNEUROSCI.17-11-04302.1997. 9151747 10.1523/JNEUROSCI.17-11-04302.1997 PMC6573547

[53] Spiridon M, Fischl B, Kanwisher N. Location and spatial profile of category-specific regions in human extrastriate cortex. Hum Brain Mapp. 2006;27:77–89. 15966002 10.1002/hbm.20169 PMC3264054

[54] Vul E, Lashkari D, Hsieh PJ, Golland P, Kanwisher N. Data-driven functional clustering reveals dominance of face, place, and body selectivity in the ventral visual pathway. J Neurophysiol. 2012;108:2306–22. 10.1152/jn.00354.2011. 22745467 10.1152/jn.00354.2011 PMC3545018

[55] Weiner KS, Grill-Spector K. The evolution of face processing networks. Trends Cogn Sci. 2015;19:240–1. 10.1016/j.tics.2015.03.010. 25840651 10.1016/j.tics.2015.03.010 PMC4414913

[56] Epstein R, Kanwisher N. A cortical representation of the local visual environment. Nature. 1998;392:598–601. 10.1038/33402. 9560155 10.1038/33402

[57] Epstein RA, Baker CI. Scene perception in the human brain. Annu Rev Vis Sci. 2019;5:373–97. 10.1146/annurev-vision-091718-014809. 31226012 10.1146/annurev-vision-091718-014809 PMC6989029

[58] Epstein RA, Julian JB. Scene areas in humans and macaques. Neuron. 2013;79:615–7. 10.1016/j.neuron.2013.08.001. 23972591 10.1016/j.neuron.2013.08.001 PMC3800114

[59] Tsitsiklis M, et al. Single-neuron representations of spatial targets in humans. Curr Biol. 2020;30:245-253 e244. 10.1016/j.cub.2019.11.048. 31902728 10.1016/j.cub.2019.11.048 PMC6981010

[60] Deen B, Koldewyn K, Kanwisher N, Saxe R. Functional organization of social perception and cognition in the superior temporal sulcus. Cereb Cortex. 2015;25:4596–609. 10.1093/cercor/bhv111. 26048954 10.1093/cercor/bhv111 PMC4816802

[61] Kosakowski HL, et al. Selective responses to faces, scenes, and bodies in the ventral visual pathway of infants. Curr Biol. 2022;32:265-274 e265. 10.1016/j.cub.2021.10.064. 34784506 10.1016/j.cub.2021.10.064 PMC8792213

[62] Pitcher D, Dilks DD, Saxe RR, Triantafyllou C, Kanwisher N. Differential selectivity for dynamic versus static information in face-selective cortical regions. Neuroimage. 2011;56:2356–63. 10.1016/j.neuroimage.2011.03.067. 21473921 10.1016/j.neuroimage.2011.03.067

[63] Weiner KS, Grill-Spector K. Neural representations of faces and limbs neighbor in human high-level visual cortex: evidence for a new organization principle. Psychol Res. 2013;77:74–97. 10.1007/s00426-011-0392-x. 22139022 10.1007/s00426-011-0392-x PMC3535411

[64] Orban GA, Sepe A, Bonini L. Parietal maps of visual signals for bodily action planning. Brain Struct Funct. 2021;226:2967–88. 10.1007/s00429-021-02378-6. 34508272 10.1007/s00429-021-02378-6 PMC8541987

[65] Urgen BA, Orban GA. The unique role of parietal cortex in action observation: functional organization for communicative and manipulative actions. Neuroimage. 2021;237:118220. 10.1016/j.neuroimage.2021.118220. 34058335 10.1016/j.neuroimage.2021.118220 PMC8285591

[66] Rolls ET. Hippocampal spatial view cells for memory and navigation, and their underlying connectivity in humans. Hippocampus. 2023;33:533–72. 10.1002/hipo.23467. 36070199 10.1002/hipo.23467 PMC10946493

[67] Maravita A, Romano D. The parietal lobe and tool use. In: Handb Clin Neurol, vol. 151. 2018. p. 481–98. 10.1016/B978-0-444-63622-5.00025-5. 10.1016/B978-0-444-63622-5.00025-5 29519476

[68] Kastner S, Chen Q, Jeong SK, Mruczek RE. B. A brief comparative review of primate posterior parietal cortex: A novel hypothesis on the human toolmaker. Neuropsychologia. 2017;105:123–34. 10.1016/j.neuropsychologia.2017.01.034. 28159617 10.1016/j.neuropsychologia.2017.01.034 PMC5537042

[69] Van Essen DC, et al. The WU-Minn Human Connectome Project: an overview. Neuroimage. 2013;80:62–79. 10.1016/j.neuroimage.2013.05.041. 23684880 10.1016/j.neuroimage.2013.05.041 PMC3724347

[70] Moeller S, et al. Multiband multislice GE-EPI at 7 tesla, with 16-fold acceleration using partial parallel imaging with application to high spatial and temporal whole-brain fMRI. Magn Reson Med. 2010;63:1144–53. 10.1002/mrm.22361. 20432285 10.1002/mrm.22361 PMC2906244

[71] Feinberg DA, et al. Multiplexed echo planar imaging for sub-second whole brain FMRI and fast diffusion imaging. PLoS One. 2010;5:e15710. 10.1371/journal.pone.0015710. 21187930 10.1371/journal.pone.0015710 PMC3004955

[72] Benjamini Y, Hochberg Y. Controlling the False Discovery Rate - a practical and powerful approach to multiple testing. J R Stat Soc B. 1995;57:289–300.

[73] Cohen J. A power primer. Psychol Bull. 1992;112:155–9. 10.1037//0033-2909.112.1.155. 10.1037//0033-2909.112.1.155 19565683

[74] Lakens D. Equivalence tests. A practical primer for t tests, correlations, and meta-analyses. Soc Psychol Personal Sci. 2017;8:355–62. 10.1177/1948550617697177. 28736600 10.1177/1948550617697177 PMC5502906

[75] Sullivan GM, Feinn R. Using effect size-or why the P value is not enough. J Grad Med Educ. 2012;4:279–82. 10.4300/JGME-D-12-00156.1. 23997866 10.4300/JGME-D-12-00156.1 PMC3444174

[76] Rolls ET, Feng J, Zhang R. Visual cortical lateralization in activations and functional connectivity to the sight of faces, scenes, body parts and tools. Hum Brain Mapp. 2026;47:e70494. 10.1002/hbm.70494. 41804040 10.1002/hbm.70494 PMC12971613

[77] Rolls ET, Turova TS. Visual cortical networks for ‘What’ and ‘Where’ to the human hippocampus revealed with dynamical graphs. Cereb Cortex. 2025;35:bhaf106. 10.1093/cercor/bhaf106. 40347158 10.1093/cercor/bhaf106

[78] Yagi S, Galea LAM. Sex differences in hippocampal cognition and neurogenesis. Neuropsychopharmacology. 2019;44:200–13. 10.1038/s41386-018-0208-4. 30214058 10.1038/s41386-018-0208-4 PMC6235970

[79] Rolls ET. Neurons in the cortex of the temporal lobe and in the amygdala of the monkey with responses selective for faces. Hum Neurobiol. 1984;3:209–22. 6526707

[80] Rolls ET, Critchley HD, Browning AS, Inoue K. Face-selective and auditory neurons in the primate orbitofrontal cortex. Exp Brain Res. 2006;170:74–87. 16328289 10.1007/s00221-005-0191-y

[81] Perrett DI, et al. Visual analysis of body movements by neurones in the temporal cortex of the macaque monkey: a preliminary report. Behav Brain Res. 1985;16:153–70. 10.1016/0166-4328(85)90089-0. 4041214 10.1016/0166-4328(85)90089-0

[82] Grill-Spector K, Weiner KS, Kay K, Gomez J. The functional neuroanatomy of human face perception. Annu Rev Vis Sci. 2017;3:167–96. 10.1146/annurev-vision-102016-061214. 28715955 10.1146/annurev-vision-102016-061214 PMC6345578

[83] Garcia-Navarra S, Solares L, Mendez M. Sex differences in the use of spatial strategies in humans: an updated systematic review of the last decade. Behav Brain Res. 2026;508:116227. 10.1016/j.bbr.2026.116227. 41997366 10.1016/j.bbr.2026.116227

[84] Marek S, et al. Reproducible brain-wide association studies require thousands of individuals. Nature. 2022;603:654–60. 10.1038/s41586-022-04492-9. 35296861 10.1038/s41586-022-04492-9 PMC8991999

[85] Cahill L. It’s time to move past biases against sex differences research: commentary on Spets and Slotnick. Cogn Neurosci. 2021;12:174–5. 10.1080/17588928.2020.1867085. 33416033 10.1080/17588928.2020.1867085

